# Physiology and Transcriptomics Analysis Reveal the Contribution of Lungs on High-Altitude Hypoxia Adaptation in Tibetan Sheep

**DOI:** 10.3389/fphys.2022.885444

**Published:** 2022-05-12

**Authors:** Pengfei Zhao, Fangfang Zhao, Jiang Hu, Jiqing Wang, Xiu Liu, Zhidong Zhao, Qiming Xi, Hongxian Sun, Shaobin Li, Yuzhu Luo

**Affiliations:** Gansu Key Laboratory of Herbivorous Animal Biotechnology, Faculty of Animal Science and Technology, Gansu Agricultural University, Lanzhou, China

**Keywords:** Tibetan sheep, lung, high-altitude hypoxia, adaptation, vascular

## Abstract

The Tibetan sheep is an indigenous species on the Tibetan plateau with excellent adaptability to high-altitude hypoxia and is distributed at altitudes of 2500–5000 m. The high-altitude hypoxia adaptation of Tibetan sheep requires adaptive reshaping of multiple tissues and organs, especially the lungs. To reveal the mechanisms of adaptation at the tissue and molecular levels in the lungs of Tibetan sheep under hypoxic conditions at different altitudes, we performed light and electron microscopic observations, transcriptomic sequencing, and enzyme-linked immunosorbent assay studies on the lungs of Tibetan sheep from three altitudes (2500, 3500, and 4500 m). The results showed that in addition to continuous increase in pulmonary artery volume, thickness, and elastic fiber content with altitude, Tibetan sheep increase the hemoglobin concentration at an altitude of 3500 m, while they decrease the Hb concentration and increase the surface area of gas exchange and capacity of the blood at an altitude of 4500 m. Other than that, some important differentially expressed genes related to angiogenesis (*FNDC1*, *HPSE,* and *E2F8*), vasomotion and fibrogenesis (*GJA4*, *FAP*, *COL1A1*, *COL1A2*, *COL3A1,* and *COL14A1*), and gas transport (*HBB*, *HBA1*, *APOLD1,* and *CHL1*) were also identified; these discoveries at the molecular level explain to some extent the physiological findings. In conclusion, the lungs of Tibetan sheep adopt different strategies when adapting to different altitudes, and these findings are valuable for understanding the basis of survival of indigenous species on the Tibetan plateau.

## Introduction

The Tibetan plateau accounts for 25% of China’s landmass and is the highest plateau on Earth with an average elevation of over 4,000 m (Thompson et al., 2000), known as “the roof of the world”’ or “the third pole.” The partial pressure of oxygen (pO_2_) decreases as the altitude increases; at an altitude of 4000 m, the pO_2_ is only 60% of that at sea level (Beall, 2014), which results in hypobaric hypoxia and poses harsh physiological challenges for indigenous species on the Tibetan plateau. Thus, the Tibetan plateau becomes an ideal natural laboratory for studying human and animal adaptation to high-altitude hypoxia. Tibetan sheep is an important domestic animal for Tibetans, which entered the Tibetan plateau 3,100 years ago from northern China through the Tang-Bo Ancient Road and settled permanently (Hu et al., 2019). Therefore, Tibetan sheep are an ideal model for studying adaptation to high-altitude hypoxia.

Animals living in the plateau show a range of physiological changes, including an increase in the alveolar surface area in Andean geese (*Chloephaga melanoptera*) (Maina et al., 2017), increased cardiac output and capillaries in the skeletal muscle in high-altitude deer mice (*Peromyscus maniculatus*) (Scott et al., 2015; Tate et al., 2020), and increased plasma volume in yaks (Bos grunniens) (Ding et al., 2014) with altitude. In addition to these, the hemoglobin (Hb) concentration increased in pigs when the altitude increased from 550 to 3300 m but decreased slightly when the altitude was over 3600 m (Kong et al., 2019), while in dogs, it increased at middle-to-high altitude compared to those at low altitude (Gou et al., 2014). In contrast to dogs, the Tibetans living at altitudes over 4300 m exhibited a similar Hb concentration to those of Han Chinese living in plains, while it was significantly lower than that of lowland sojourners at altitudes over 4300 m. For Andeans living at altitudes over 4300 m, the Hb concentration similar to the lowland sojourners at the same altitude was exhibited significantly higher (Beall, 2007; Peng et al., 2017). This implies that different high-altitude indigenous species or populations have different mechanisms for adapting to high-altitude hypoxia. Other than the almost constant Hb concentration, native Tibetans acquired better ventilation efficiency and larger lungs (Wu and Kayser, 2006) and exhibited significantly increased plasma volume than lowland sojourners and Andeans at similar altitudes (Stembridge et al., 2019). This suggests that Tibetans did not have a blunted physiological response to hypoxia but maintained a high level of pulmonary blood gas exchange by increasing the volume of the lungs and total Hb mass adaptation to high-altitude hypoxia. These results emphasize the importance of integrating studies of multiple traits of adaptation to high-altitude hypoxia rather than highlighting the single trait in isolation.

Regarding sheep, Wei et al. (2016) found that the Hb concentration and hematocrit (Hct) of Tibetan sheep at an altitude of 3000 m were significantly higher than those of large-tailed Han sheep at an altitude of 100 m, and Wang et al. (2019) found that the resting respiratory rate was significantly higher in Tibetan sheep at an altitude of 3500 m than in small tail Han sheep at an altitude of 1500 m. These studies on sheep are between two breeds at two altitudes, and they focused on few traits associated with high-altitude hypoxia adaptation. There is no evidence that these traits are always linearly related to altitude, and high-altitude hypoxia adaptation is a complex reciprocal process of multiple traits. Therefore, a simple comparison of two altitudes and observation of few traits cannot fully reveal the complex adaptation mechanism of Tibetan sheep to different altitudes, and different breeds bring unnecessary errors into the study results. In recent years, transcriptomics have been used to study hypoxia adaptation of Tibetan indigenous animals (Zhang et al., 2015; Jia et al., 2016; Li et al., 2017; Qi et al., 2019; Xin et al., 2019), but each of these studies had their own imperfection, either involving only one or two altitudes or between different breeds or focusing on a few phenotypic traits associated with high-altitude hypoxia.

**FIGURE 1:**
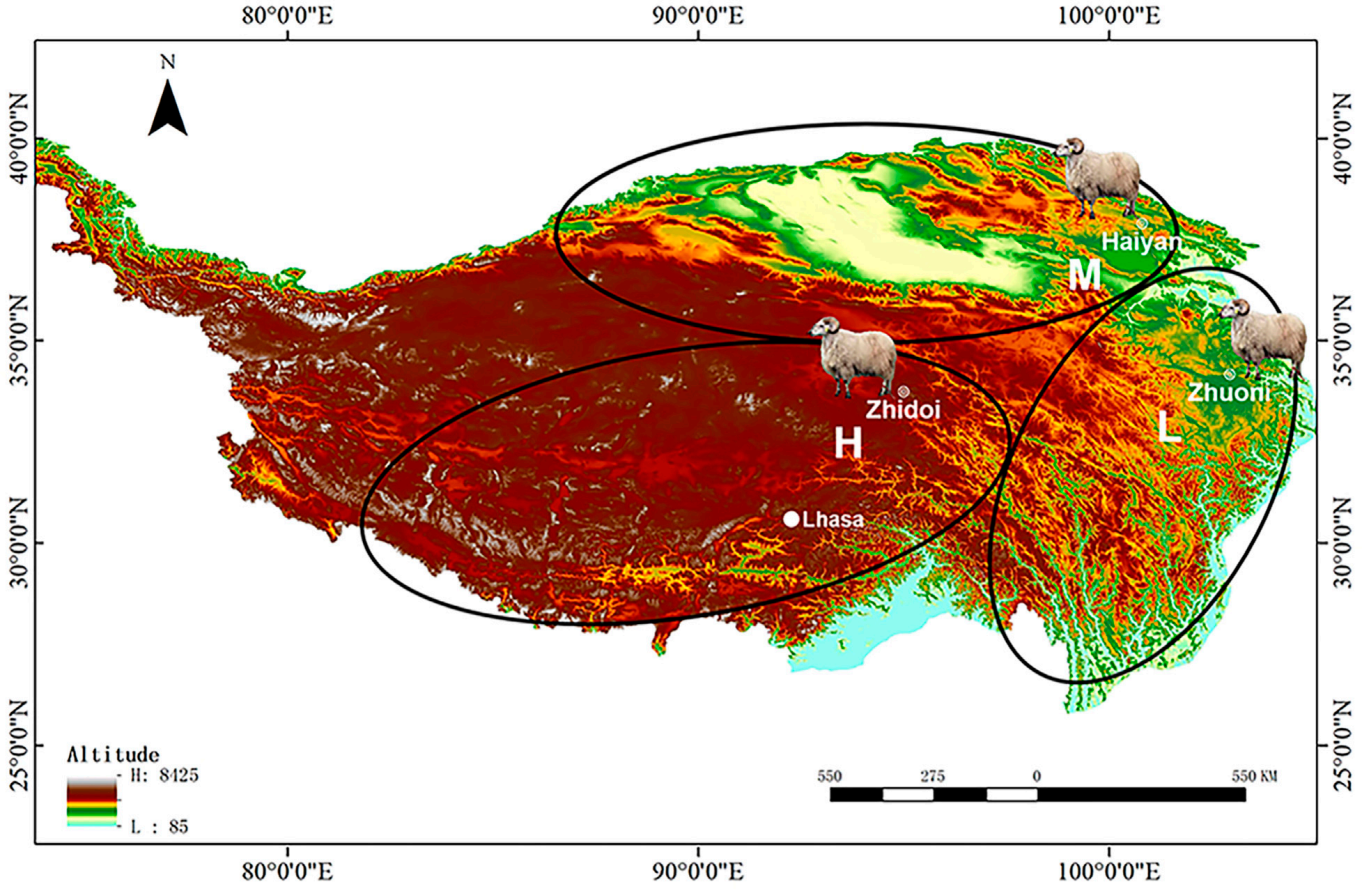
Sampling sites of Tibetan sheep from different altitude distribution areas on the Tibetan plateau. The 2500-m groups from the “low-altitude” region at Zhuoni County, Gannan Tibetan Autonomous Prefecture, China; the 3500-m groups from the “middle-altitude” region at Haiyan County, Haibei Tibetan Autonomous Prefecture, China; the 4500-m groups from the “high-altitude” region at Zhidoi County, Yushu Tibetan Autonomous Prefecture, China. L, M, and H represented low-, middle-, and high-altitude regions, respectively.

In order to comprehensively understand whether Tibetan sheep at different altitudes have different adaptation mechanisms to hypoxia, this study selected Tibetan sheep at three altitudes (2500, 3500, 4500 m) according to their three main distribution areas on the Tibetan plateau (Figure 1) and observed their lung structures using optical and electron microscopes, screened key genes in their lungs associated with high-altitude hypoxia using RNA-seq, and the expression levels of key protein were measured using enzyme-linked immunosorbent assay (ELISA). By leveraging the information of physiology and transcriptomics from multiple altitudes, this study found that the phenotypic changes and a set of genes and molecular pathways may explain how the lungs contribute to the high-altitude hypoxia adaptation of Tibetan sheep.

## Materials and Methods

### Sheep Investigated and Sample Collection

Tibetan sheep can be divided into three main distribution areas according to the altitude from low to high: southern Tibet valley and eastern fringe of the Tibetan plateau, vast area surrounding the Qaidam Basin, southern Qinghai province, and the Tibetan hinterland. Based on this, six healthy ewes approximately 3 years old at low (L), middle (M), and high (H) altitudes of approximately 2500, 3500, and 4,500 m were selected for this study, respectively (Figure 1). After weighing live, sheep were slaughtered and the wet weight of the heart and lung was weighed; then, the parenchyma of the right lung was quickly collected for RNA-seq, ELISA, transmission electron microscopy (TEM) observation, hematoxylin–eosin (HE), and the Weigert resorcinol magenta (WRM) staining, and the left lung was collected for preparing artery corrosion cast and it was then observed using scanning electron microscopy (SEM).

### Histological Analysis of the Lungs

Histological analysis includes TEM observation and HE and WRM staining. TEM can visualize the ultrastructure of cells, which is valuable in analyzing cellular components (CCs) such as organelles, cytoskeleton, and membrane systems (Winey et al., 2014) and is widely used in the field of cell biology (Delavoie et al., 2021; Malatesta, 2021). In this study, one sample per group was used for TME observation; the samples were fixed with 3% glutaraldehyde at 4°C, refixed with 1% osmium tetroxide, then dehydrated in graded acetone (30%→50%→70%→80%→90%→95%→100%; 10 min each and three changes in 100% concentration), and successively soaked with acetone and epoxy resin in the ratio of 3:1, 1:1, and 1:3 for 40 min per step. Then, the samples were embedded in epoxy resin, dried, and cut into ultrathin sections using an EM-UC7 microtome (Leica, Wetzlar, Germany); the sections were stained with uranyl acetate and lead citrate for 15–20 min, and the ultrastructure of the alveolar septum was observed by a JEM-1400PLUS TEM (JEOL, Tokyo, Japan).

Hematoxylin can stain the nucleus purple–blue and eosin can stain the cytoplasm and extracellular matrix (ECM) red, making the tissue structure clearly visible (Wittekind, 2003). WRM can dye the elastic fibers dark blue, making the widely distributed elastic fibers in the walls of alveoli and blood vessels be visualized (Proctor and Horobin, 1988). Both staining techniques are widely used in histological and pathological studies (Hochstim et al., 2010; Hernandez-Morera et al., 2017). In this study, three and one samples per group were used for HE and WRM staining, respectively; the samples were fixed with 4% paraformaldehyde, then dehydrated in graded ethanol (75%→85%→95%→100%), rinsed in xylol, and embedded in paraffin. Then, the embedded samples were cut into 5-µm thick sections using a Leica-2016 rotary microtome (Leica, Wetzlar, Germany); the HE and WRM staining was performed by Lilai Biotech Co., Ltd. (Chengdu, Chain). Three different 100x micrograph fields of view of HE-stained sections and three different 400x micrograph fields of view of WRM-stained sections were taken by BA210 Digital microscope camera (Motic, Xiamen, China) for arteriole count and area measurement and elastic fiber observation.

### Corrosion Cast Making of Pulmonary Artery and SEM Observation

Corrosion cast has an important place in anatomical studies, as it clearly reflects the branching and distribution of blood vessels and secretory ducts (Pálek et al., 2018), and SEM can observe the fine features on the surface of the corrosion cast (Lametschwandtner et al., 1990). In this study, 100 and 150 g of acrylonitrile–butadiene–styrene plastic pellets were dissolved in 1000 ml of a 1:1 mixture of acetone and butanone containing 1 g of Sudan Ⅲ dye to prepare approximately 10 and 15% of the casting agent, respectively. From the aorta of the left lung, 10 and 15% of the casting agent was injected successively, and the perfusion was completed when the artery was full. After the casting agent was fully solidified, the left lung was placed in 30% hydrochloric acid and corroded for 10–12 days to obtain the pulmonary artery corrosion cast. Arteriole branches in the same area of each specimen were selected, cleaned with an ultrasound, dried, then sprayed with gold using an E-1045 ion coater (Hitachi, Tokyo, Japan), and observed by an S-3400N SEM (Hitachi, Tokyo, Japan).

### RNA-sequencing and Data Analysis

Four lungs from four Tibetan sheep per altitude were used for RNA-Seq; the quickly collected samples were frozen immediately in liquid nitrogen. Total RNA was isolated using TRIzol reagent (Invitrogen, Carlsbad, CA, United States), and RNA quality was assessed using an Agilent 2100 Bioanalyzer (Agilent, Palo Alto, CA, United States). Libraries of cDNA were generated using the TruSeq RNA Sample Preparation Kit (Illumina, San Diego, CA, United States) and then sequenced on the Illumina Novaseq6000 (Illumina, San Diego, CA, United States) platform by Gene Denovo Biotech Co., Ltd. (Guangzhou, China).

Raw reads from the sequencing machines are stored in FASTQ format, and clean reads were obtained by removing the reads containing adapters; more than 10% of unknown nucleotides and quality scores < Q20 (the proportion of read bases whose error rate is less than 1%) were obtained using fastp v0.18.0 (Chen et al., 2018). Then, Bowtie2 v2.2.8 (Langmead and Salzberg, 2012) was used for mapping clean reads to the rRNA database, and mapped reads were removed. The remaining clean reads were mapped to the ovine genome assembly Oar_rambouillet_v1.0^1^ using HISAT2 v2.4.0 (Kim et al., 2015). Gene expression abundance was normalized by the fragment per kilobase of transcript per million (FPKM) mapped reads, and FPKM >0.01 was expressed (Trapnell et al., 2010). The differentially expressed gene (DEG) identification was performed by DESeq v2.0 (Love et al., 2014) between the samples of different altitudes, and genes with a false discovery rate (FDR) < 0.05 and a fold change ≥2 or ≤0.5 were considered DEGs.

### GO Enrichment, KEGG Pathways, and Cluster Analysis of the DEGs

The Gene Ontology (GO) database^2^ was used to perform the functional enrichment analysis of the DEGs; the biological process (BP), cellular component (CC), and molecular function (MF) were annotated for the DEGs. Pathway analysis of the DEGs was performed using the Kyoto Encyclopedia of Genes and Genomes (KEGG) database^3^. The significantly enriched GO terms and KEGG pathways (*p* < 0.05) were defined using Fisher’ exact and χ^2^ tests.

In order to examine the expression pattern of DEGs, the fold change for all samples was calculated relative to the low-altitude group and then classified by short time-series expression miner (STEM) (Ernst and Joseph, 2006) software. The output profile number was eight, and the profiles with *p* < 0.05 were regarded as significant profiles.

### Validation of RNA-seq Results by RT-qPCR

To validate the reliability of the RNA-seq results, reverse transcription-quantitative PCR (RT-qPCR) analysis was conducted on 12 randomly selected DEGs. These DEGs and their primer information are listed in Table 1; the gene *GAPDH* was used as an internal reference to normalize the expression level of these DEGs. PCR primers of these genes were designed using primer 5.0 and synthesized by the Takara Biotech Co., Ltd. (Dalian, China). The RNA samples used for RNA-seq are also used to synthesize cDNA using the SuperScript TM II reverse transcriptase (Invitrogen, Carlsbad, CA, United States), and RT-qPCR was performed in four replicates using a 2×ChamQ SYBR qPCR Master (Vazyme, Nanjing, China) on an Applied Biosystems QuantStudio^®^ six Flex (Thermo Lifetech, Waltham, CA, United States). The relative expression levels of these genes were calculated by the 2^−ΔΔCt^ method.

**TABLE 1:** Primers used for the RT-qPCR analyses.

Gene	Forward (5’→3′)	Reverse (5’→3′)
*ACTG2*	GTG​ACA​TTG​ACA​TCC​GCA​AG	CTG​CTG​GAA​GGT​GGA​GAG​AG
*EHD3*	TTC​ACG​CCT​ACA​TCA​TCA​GC	TAG​CTG​GTC​CTG​CAT​CCT​CT
*SPARC*	TTC​GAC​TCT​TCC​TGC​CAC​TT	TTG​TTG​TCC​TCG​TCC​CTC​TC
*MEGF6*	ATG​CTT​CCT​TCC​CTG​TCC​TT	TGG​AAA​AGA​CCC​TGA​TCC​TG
*MMP12*	GGA​CCC​TGG​TTA​TCC​CAA​GT	ATG​ACA​CGT​TGG​GCT​AGG​AC
*ASB2*	AGT​ACA​AGG​CGG​ACA​CCA​AC	CAC​CAG​GAT​CTC​CAT​GAC​CT
*COL3A1*	GGT​CCA​GGA​TTG​AGG​GGT​AT	GGT​CCA​GGA​TTG​AGG​GGT​AT
*PI16*	AGG​AGA​CCG​ACA​TCC​ACT​TG	CCA​GGG​AAG​ACA​AAT​CCT​GA
*NR1D1*	GCT​TGG​GTG​ATG​ATG​GTT​CT	GCT​CTT​GCT​GGG​AGA​CAC​TC
*COL1A1*	AGC​CAG​CAG​ATC​GAG​AAC​AT	TGG​GGT​ACA​CAC​AGG​TCT​CA
*E2F8*	ACT​ATC​CCA​ACC​CTG​CTG​TG	AGG​GTC​TGG​TTG​AGG​TTG​TG
*HBB*	CTT​GTC​CTC​TGC​TGA​TGC​TG	ACC​AGT​ATG​TTG​CCC​AGG​AG

### Quantification of Key Protein HBB by ELISA

*HBB* is the key gene screened by RNA-seq, and the abundance of HBB protein was further determined by ELISA in this study. Six Tibetan sheep per altitude were used; 100 mg of the lung parenchyma per sheep was triturated by an F6/10-10G ultrafine homogenizer (Fluko, Shanghai, China) and then mixed with 500 µL PBS. The solution was centrifuged at 4°C for 10 min at 3,000×g, and the supernatant was collected. According to the instruction of the corresponding sheep ELISA kit (Zcibio, Shanghai, China), the absorbance of HBB protein at 450 nm was measured, and the concentration of HBB was calculated from the corresponding HBB standard curve.

### Statistical Analyses

The number and average area of arterioles in each field of view of HE-stained sections were counted and measured using Image-Pro Plus 6.0. Three most desirable corrosion casts were selected from each altitude, and their aorta and first-, second-, third-, and fourth-grade branch diameters were measured at the root, middle, and terminal three positions using a digital Vernier caliper, and the average value was calculated.

One-way analysis of variance (ANOVA) was performed using SPSS 19.0 on the proportion of the lung and heart to body weight, number, and area of arterioles; on the proportion of aorta and first-, second-, third-, and fourth-grade branch diameters to lung weight (mm/g); and ELISA results between different altitudes. This was expressed as mean ± SD; the statistical significance was set at *p* < 0.05 and was extremely significant at *p* < 0.01.

## Results

### Physiological Differences in the Lungs

**FIGURE 2:**
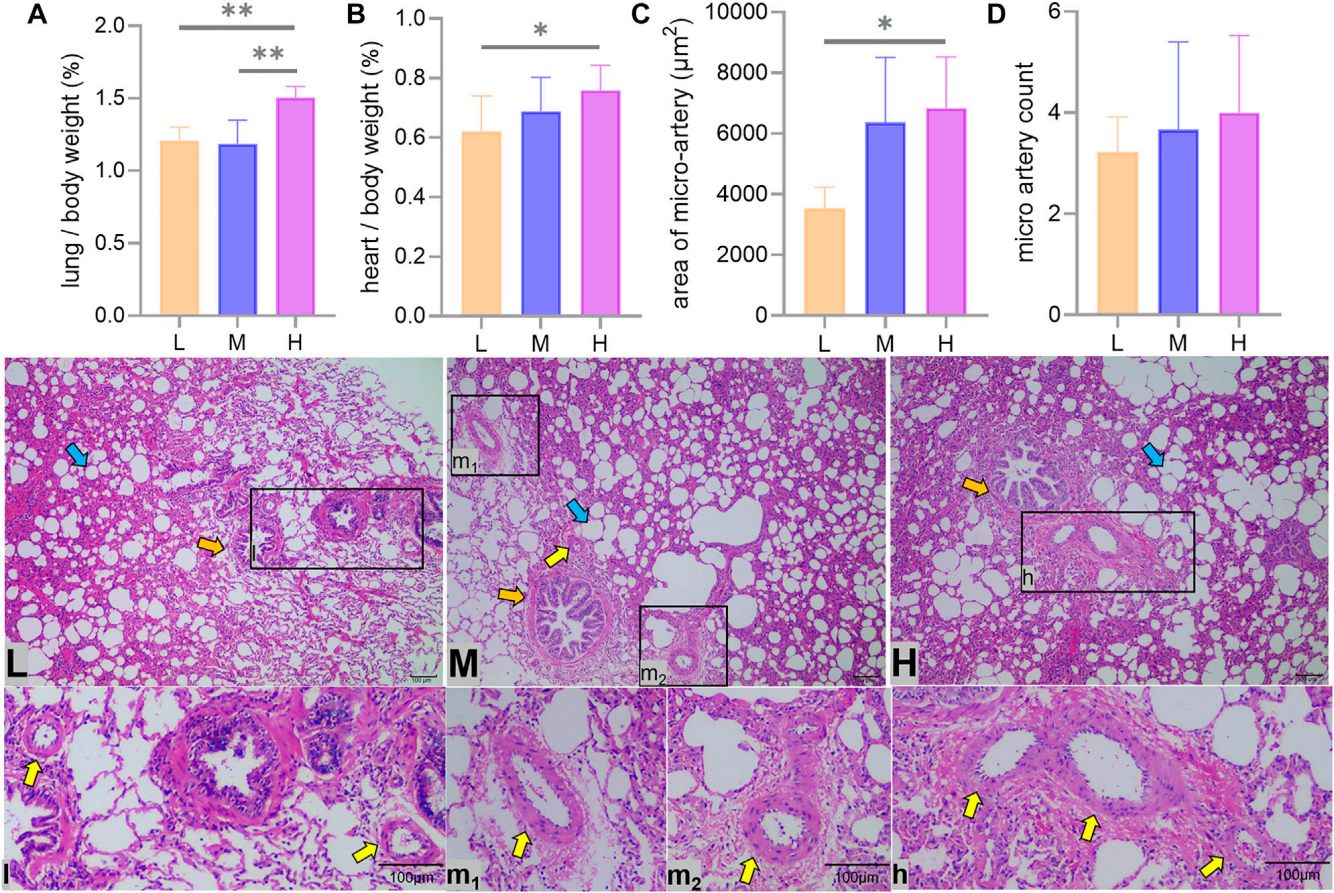
Heart and lung to body weight ratio and lung hematoxylin–eosin staining. Differences in lung **(A)** and heart **(B)** as a percentage of body weight, average area **(C),** and number **(D)** of arterioles at a 100x field of view between low (L), middle (M), and high (H) altitudes and arterioles under optical microscope are shown. * and ** indicate significant (*p* < 0.05) and extremely significant (*p* < 0.05) differences between different altitudes, respectively; yellow arrows indicate arterioles, orange arrows indicate terminal bronchioles, and blue arrows indicate alveoli.

The proportion of lungs to body weight in Tibetan sheep hardly changed from L to M altitude (*p* > 0.05), and it showed an increase from M to H altitude (*p* < 0.01) (Figure 2A), and the proportion of heart to body weight gradually increased with altitude (*p* < 0.05) (Figure 2B).

The average area of the arterioles measured showed an increase from L to H altitude (*p* < 0.05) (Figures 2C,l,m_1,_m_2_,h), and the number of arterioles tended to increase with altitude, but the difference was not significant (*p* > 0.05) (Figure 2D).

**FIGURE 3:**
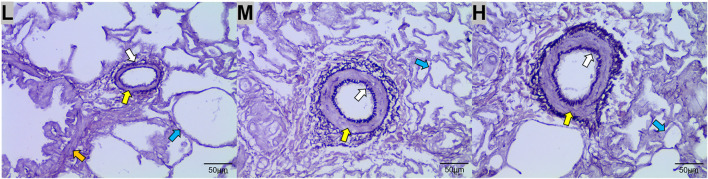
Weigert resorcinol magenta (WRM) staining of the lungs of Tibetan sheep at low **(L)**, middle **(M),** and high **(H)** altitudes at 400x field of view. Yellow arrows indicate arterioles, orange arrows indicate terminal bronchioles, blue arrows indicate alveoli, and white arrows indicate elastic fibers.

The results of WRM staining showed that the walls of the lung arterioles thickened and the elastic fiber content increased with increasing altitude (Figure 3).

**FIGURE 4:**
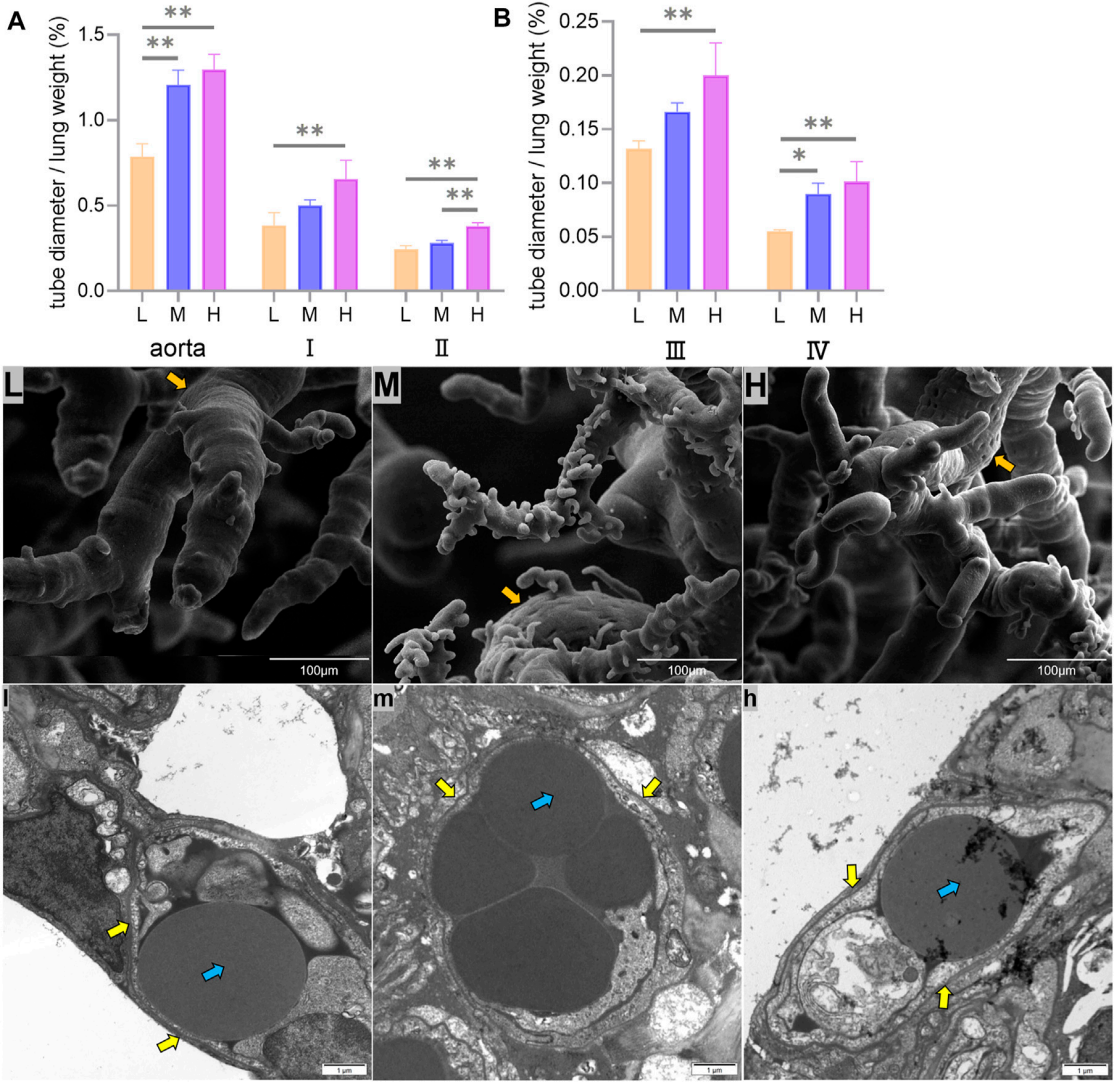
Differences in the ratio of the aorta and its first-, second-, third-, and fourth-grade branch diameters to the weight of the lung (mm/g) among low (L), middle (M), and high altitudes **(A,B)**, surface characteristics of corrosion casts under the 800x field of view of the scanning electron microscopy (SEM) **(L,M,H)**, and capillary artery ultrastructure under the 12000x field of view of the transmission electron microscopy (TEM) **(l,m,h)** are shown. * and ** indicate significant (*p* < 0.05) and extremely significant (*p* < 0.05) differences between different altitudes, respectively; yellow arrows indicate vascular endothelial cells, orange arrows indicate endothelial cell indentations, and blue arrows indicate erythrocyte.

The left lung aorta and its first-, second-, and third-grade branches with diameters greater than 0.5 mm are elastic arteries, while the fourth-grade branches with diameters less than 0.5 mm are muscular arteries. The ratio of the artery tube diameter to the weight of the lung in Tibetan sheep was used to represent the vascular volume, and it was found that the vascular volume of all branches increased with altitude (*p* < 0.01) (Figures 4A,B).

Fine features were observed on the surface of the pulmonary artery corrosion cast under the 800x field of view using SEM, and it was found that the endothelial cell indentations became deeper and more numerous with increasing altitude (Figures 4L,M,H). The observation of capillary arteries using TEM under 12000x field of view revealed an increase in the volume and muscularization of vascular endothelial cells with increasing altitude (Figures 4l,m,h).

### Summary of the RNA-seq Data

Twelve separate cDNA libraries were constructed from the lung tissue of Tibetan sheep (four each from L, M, and H altitude). These cDNA libraries were used for RNA-seq, and the summary of the RNA-seq data is shown in Table 2. On average, 41,663,147, 41,502,286, and 41,379,484 raw reads were produced from L, M, and H altitude, respectively. After filtering the reads that contain adapters, more than 10% of unknown nucleotides and quality scores < Q20, an average of 40,961,355, 40,882,558, and 40,907,642 clean reads was obtained from the three altitudes, respectively. The correlations between the four samples within the L, M, and H altitude were above 0.97, 0.95, and 0.94, respectively. After removing the reads that mapped to rRNA, the remaining clean reads were 40,835,044, 40,836,250, and 40,865,366, respectively. Of the remaining clean reads, an average of 39,094,467 (95.74%), 39,215,051 (96.03%), and 39,244,838 (96.04%) reads was well-mapped to the ovine genome assembly (Oar_rambouillet_v1.0), with a unique match ratio of 89.03, 89.72, and 90.17%, respectively. Using a threshold of FPKM >0.01 to define the potentially expressed genes, a total of 17,332, 17,369, and 17,460 expressed genes were detected in the lung tissues of Tibetan sheep from L, M, and H altitude, respectively, with 16,585 genes being expressed at all three altitudes.

**TABLE 2:** Summary of the RNA-seq data.

Samples	Average raw reads	Average clean reads	Average remaining clean reads	Average mapped reads	Average unique reads	Average multiple reads
L	41,663,147	40,961,355	40,835,044	39,094,467 (95.74%)	36,355,386 (89.03%)	2,739,080 (6.71%)
M	41,502,286	40,882,558	40,836,250.5	39,215,051 (96.03%)	36,638,505 (89.72%)	2,576,545 (6.31%)
H	41,379,484	40,907,642	40,865,366	39,244,838 (96.04%)	36,847,114 (90.17%)	2,397,724 (5.87%)

**FIGURE 5:**
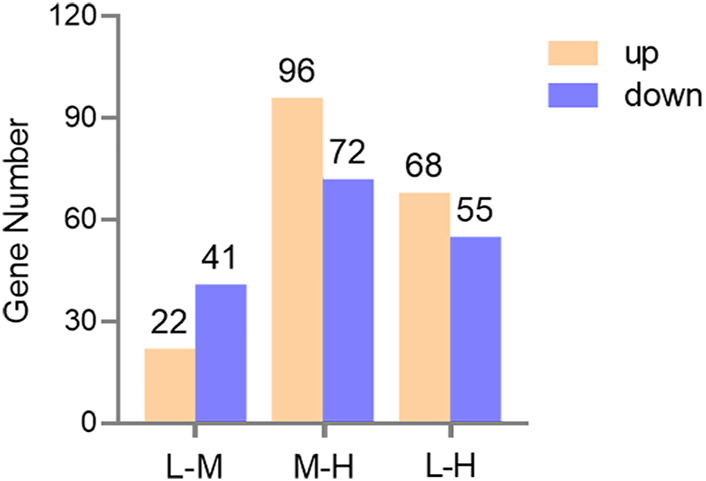
Bar graph of the DEGs in three comparisons. Yellow and blue represent upregulated and downregulated gene numbers, respectively.

A total of 63, 168, and 123 genes were identified to be differentially expressed when comparing L and M, M and H, and L and H (FDR <0.05 and fold change ≥2 or ≤0.5), respectively. Of these, 22, 96, and 68 genes were significantly upregulated and 41, 72, and 55 genes were significantly downregulated (Figure 5).

### GO Enrichment Analysis of the DEGs

**FIGURE 6:**
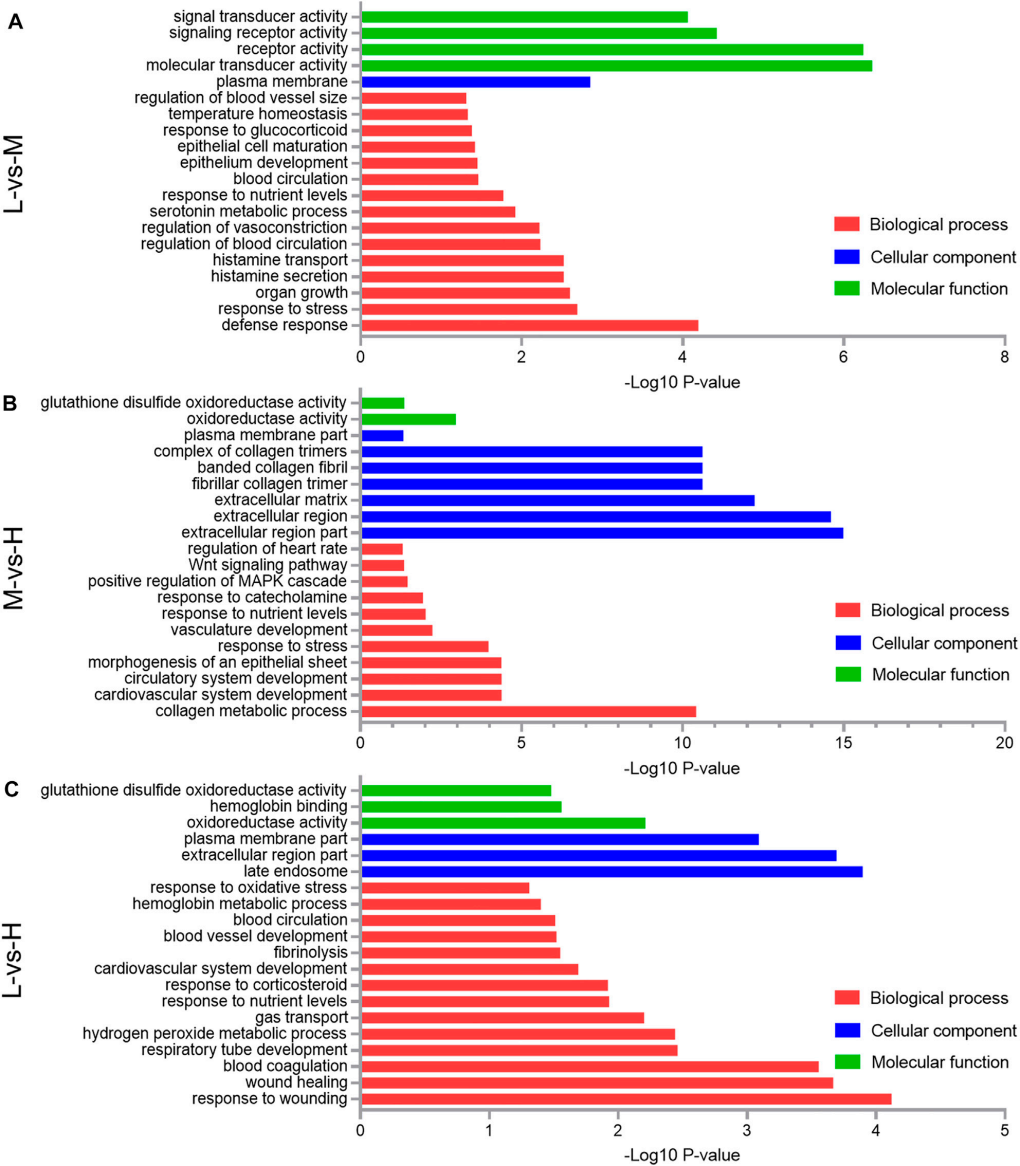
Gene Ontology (GO) classification of the differentially expressed genes (DEGs) comparing the L and M **(A)**, M and H **(B)**, and L and H **(C)**. A portion of the significantly enriched biological process, cellular component, and molecular function GO terms is shown.

Concerning the 63 DEGs between L and M, they were significantly enriched in 155 BP terms, 16 CC terms, and 18 MF terms. The top five significant terms with the lowest *p*-value were molecular transducer activity (*p* = 4.47E-07), receptor activity (*p* = 5.75E-07), signaling receptor activity (*p* = 3.8E-05), defense response (*p* = 6.4E-05), and signal transducer activity (*p* = 8.7E-05); in addition to these, important GO terms related to blood circulation (*p* = 0.035) and regulation of blood vessel size (*p* = 0.049) were also found (Figure 6A).

Concerning the 168 DEGs between M and H, they were significantly enriched in 477 BP terms, 28 CC terms, and 50 MF terms. The top five significant terms with the lowest *p*-value were extracellular region part (*p* = 1.05E-15), extracellular region (*p* = 2.51E-15), extracellular matrix (*p* = 5.89E-13), fibrillar collagen trimer (*p* = 2.45E-11), and banded collagen fibril (*p* = 2.45E-11); in addition to these, important GO terms related to the collagen metabolic process (*p* = 3.8E-11) and vasculature development (*p* = 0.006) were also found (Figure 6B).

Regarding the 123 DEGs between L and H, they were significantly enriched in 201 BP terms, 27 CC terms, and 54 MF terms. The top five significant terms with the lowest *p*-value were response to wounding (*p* = 7.6E-05), late endosome (*p* = 0.00013), extracellular region part (*p* = 0.0002), wound healing (*p* = 0.00022), and blood coagulation (*p* = 0.00028); in addition to these, important GO terms related to gas transport (*p* = 0.0063) and blood vessel development (*p* = 0.03) were also found (Figure 6C).

### KEGG Pathway Analysis of the DEGs

**FIGURE 7:**
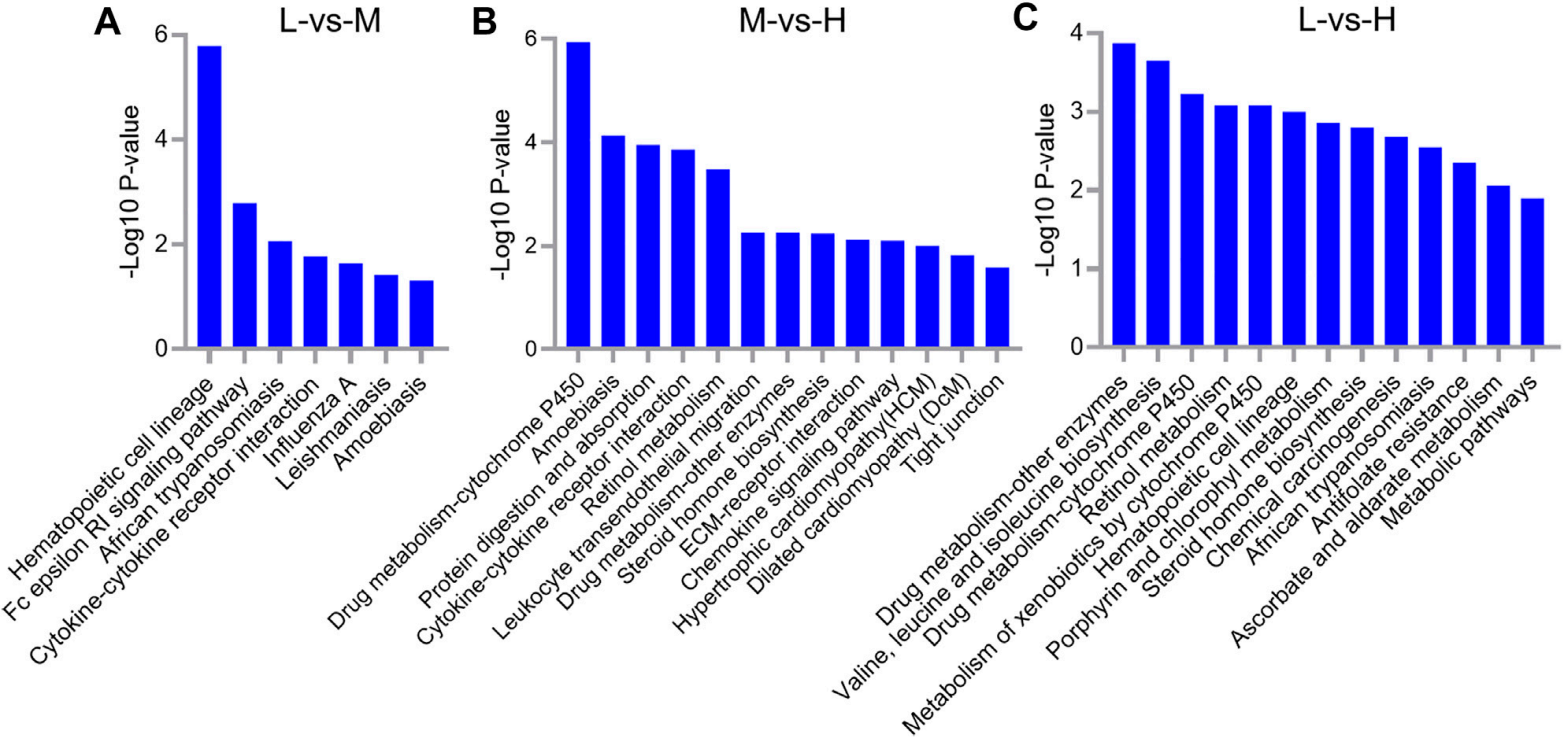
KEGG pathway analysis for differentially expressed genes (DEGs) between L and M **(A)**, M and H **(B)**, and L and H **(C)**. A portion of the significantly enriched pathways is shown.

The KEGG analysis of the 63 DEGs between L and M showed that hematopoietic cell lineage (*p* = 1.62E-06), Fc epsilon RI signaling pathway (*p* = 0.0016), African trypanosomiasis (*p* = 0.0085), and cytokine–cytokine receptor interaction (*p* = 0.017) were the top four enriched pathways (Figure 7A). Concerning the 168 DEGs between M and H, the top four enriched pathways were drug metabolism–cytochrome P450 (*p* = 1.15E-06), amebiasis (*p* = 7.41E-05), protein digestion and absorption (*p* = 0.00011), and cytokine–cytokine receptor interaction (*p* = 0.00014); in addition to these, the important pathway ECM–receptor interaction (*p* = 0.0077) was also identified (Figure 7B). Concerning the 123 DEGs between L and H, the top four enriched pathways were drug metabolism–other enzymes (*p* = 0.00013), valine, leucine, and isoleucine biosynthesis (*p* = 0.00022), drug metabolism–cytochrome P450 (*p* = 0.00059), and retinol metabolism (*p* = 0.00083) (Figure 7C).

### Cluster Analysis of the DEGs

**FIGURE 8:**
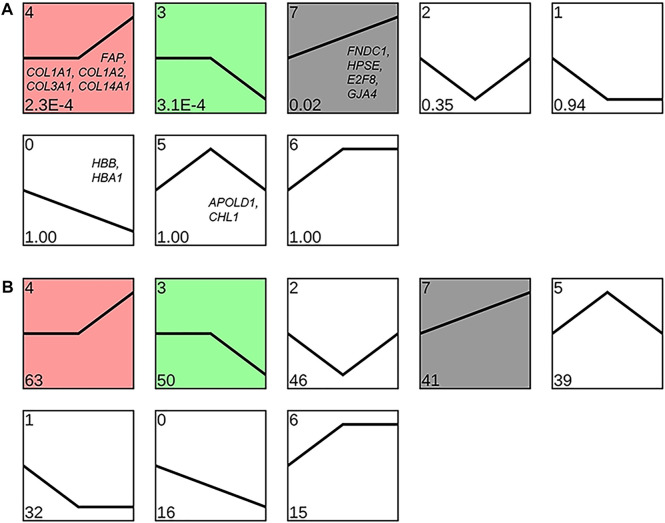
Clustering analysis of the expression pattern of differentially expressed genes (DEGs) in Tibetan sheep lungs with increasing altitude. Expression patterns are shown based on the *p*-value **(A)** and number of DEPs **(B)** in the lower left corner; the genes in the patterns are the key DEGs enriched in the corresponding patterns.

Clustering analysis of the expression patterns of DEGs yielded eight different expression patterns, three of which were significantly enriched in the lungs of Tibetan sheep with increase in altitude (*p* < 0.05). Profile 4 and profile 7 showed similar expression patterns, with a significant upregulation of DEGs during an increase in altitude, while profile 3 showed a significant downregulation of DEGs during an increase in altitude (Figure 8).

### Validation of RNA-seq Results by RT-qPCR and Quantification of HBB Protein

**FIGURE 9:**
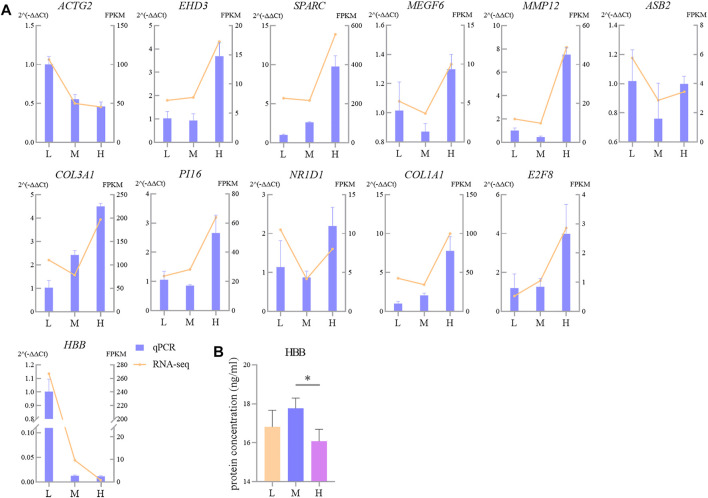
Comparison of gene expression levels measured using RNA-seq and RT-qPCR for 14 randomly selected DEGs **(A)** and protein abundance of HBB **(B)**. L, M, and H refer, respectively, to low, middle, and high altitudes; the RT-qPCR and ELISA results are presented as mean ± SD for four and six replicates, respectively; * indicates *p* < 0.05.

Twelve randomly selected DEGs were used to perform RT-qPCR; the results were congruent with the RNA-seq data (Figure 9A). It indicates that the quantification of gene expression in Tibetan sheep lungs by RNA-seq is reliable and repeatable. The HBB protein was quantified by ELISA, and the results showed that the concentration of HBB protein in the lungs gradually increased as the altitude increased from L to M; when the altitude increased further to H, it decreased significantly (*p* < 0.05) (Figure 9B).

## Discussion

The weight of the lungs and heart of Tibetan sheep may be related to altitude. The previous studies found that the Quechua women at high altitude have larger lung volumes than their plain counterparts (Kiyamu et al., 2012), and the heart of yak is larger than that of cattle (Wang et al., 2016). In this study, six Tibetan sheep of similar body condition and good health were selected from each altitude and weighed for pre-slaughter live weight and wet weight of heart and lungs, and it was found that the proportion of lung to body weight was greater (*p* < 0.01) in Tibetan sheep at H altitude than their counterparts at L and M altitude (Figure 2A), and the proportion of heart to body weight of Tibetan sheep increased (*p* < 0.05) with altitude (Figure 2B). Since these sheep were in similar body condition and in good health, it can be assumed that the difference in the heart and lung to body weight ratio between altitudes was mainly due to the difference in oxygen concentration. The larger lung and heart required an enlarged thorax to accommodate, which was found in humans and yaks that inhabited high altitudes (Wiener et al., 2003; Penaloza and Stella, 2007). Larger lungs facilitate increased ventilation, allowing for more oxygen to be dissolved in pulmonary arterial blood, and a larger heart can pump oxygen-rich arterial blood more efficiently throughout the body. At high altitude, Tibetan sheep have a higher respiratory rate (Wang et al., 2019), the Han Chinese sojourners have an increased heart rate with altitude (Rao et al., 2015), and the Tibetans have higher pulmonary ventilation (Bigham and Lee, 2014). Higher pulmonary ventilation may be underlain by large thorax and lung volumes and high respiratory rates; similarly, large thorax and heart volumes and high heart rates may result in high cardiac output. The high pulmonary ventilation and cardiac output based on large lung and heart may be the result of adaptation to hypoxic environment in animals living at high altitude.

When cardiac output increases with altitude, the pulmonary artery of Tibetan sheep requires having powerful vasodilatation and vasoconstriction capacity to avoid pulmonary hypertension. This study found that the thickness of the pulmonary artery of Tibetan sheep increased with altitude (Figures 3L,M,H). Similarly, a study found that hypoxia promotes vascular remodeling (Maron et al., 2014), leading to muscularization of the human pulmonary vasculature (Wilkins et al., 2015). In addition, Wang et al. (2013) found that hypoxia damages the integrity of endothelial cell and triggers the inflow of growth factors, leading to smooth muscle cell proliferation and pulmonary artery thickening. A thicker pulmonary artery contains more smooth muscle and elastic fibers, which can accommodate more blood by vasodilatation when the right ventricular systolic delivers blood to the whole lung by vasoconstriction. Observation of the pulmonary artery corrosion casts by SEM showed deeper and denser indentations of vascular endothelial cells on the surface with increasing altitude (Figures 4L,M,H); this suggests that thickened pulmonary arteries in a hypoxic environment have strong contractile capacity. Consistent with this study, Yang et al. (2021) found that the indentations in corrosion casts were deeper and denser in the pulmonary artery of Tibetan pigs than in lower-altitude pig breeds. Observation of the alveolar wall vessels by TEM revealed that with increasing altitude, the alveolar wall vessels became muscularized and the endothelial cells became hypertrophic (Figures 4l,m,h); this muscularization was also found in many animal models under hypoxic environments such as the rat (Stenmark et al., 2006). In summary, the thickened pulmonary artery allows Tibetan sheep to better control the flow of blood in the lungs under hypoxic environment, thus avoiding pulmonary hypertension.

Adequate blood oxygen exchange requires not only high pulmonary ventilation and artery vasodilatation and vasoconstriction ability but also large vascular volume. In this study, the ratio of the left lung artery and its first- to fourth-grade branch diameters to lung weight, which was a marker of pulmonary artery volume size, was found to increase with altitude (*p* < 0.01) (Figures 4A,B). This study also found that the average cross-sectional area of arterioles of the lungs was higher (*p* < 0.05) in Tibetan sheep at H altitude than their counterparts at L altitude (Figure 2C), while the number of arterioles did not differ significantly between altitudes (*p* > 0.05) (Figure 2D). Although the cross-sectional area of arterioles did not differ significantly between M and H altitudes (*p* > 0.05), the ratio of lungs relative to body weight was greater (*p* < 0.01) at H altitude than at M altitude, suggesting that Tibetan sheep at H altitude have a higher arteriole volume. Consistent with this study, Maina et al. (2017) found that the lungs of Andean geese living at altitudes of 3,000–5,500 m were intensely vascularized. These morphological changes in the lungs of Tibetan sheep and Andean geese not only increase the surface area for gas exchange but also increase the capacity of the blood. A larger capacity of the blood reduces peripheral resistance to avoid pulmonary hypertension, and the thickened pulmonary artery delivers blood throughout the lungs by elastic recoil for adequate blood oxygen exchange with abundant oxygen provided by high pulmonary ventilation. This may be the cardiorespiratory basis of adaptation to high-altitude hypoxia in Tibetan sheep.

In order to reveal the molecular mechanisms by which the lungs of Tibetan sheep exhibit different morphological and histological characters at different altitudes, this study performed RNA-seq for 12 lungs at three altitudes. A total of 302 DEGs were identified between the three altitudes; the number of DEGs between L and H and M and H altitudes was greater than that between L and M altitudes, which suggests that the lungs of Tibetan sheep have different gene expression patterns between L and H and M and H altitudes, which is consistent with the results of morphologically observation in this study.

Among the DEGs between L and H altitudes, *FNDC1*, *HPSE*, *E2F8*, *GJA4*, and *FAP* were the most prominent upregulated genes at H altitude. *FNDC1*, *HPSE*, and *E2F8* were associated with angiogenesis, and *FNDC1* is involved in the vascular endothelial growth factor (VEGF)–induced angiogenesis. Angiogenesis is inhibited when *FNDC1* is knocked down using small-interfering RNA (Hayashi et al., 2016). Increased vascularity was observed after the local administration of *HPSE*-encoded heparinase in a mouse wound healing study (Elkin et al., 2001). *E2F8* activates *VEGF* transcription, and the knockout of *E2F8* in mice develops severe vascular defects at the embryonic stage (Weijts et al., 2014). The expression patterns of these three genes were clustered into profile 7 (Figure 8A); therefore, it is hypothesized that the thickening of the pulmonary artery in Tibetan sheep with altitude (Figure 3) is due to the increased expression of these three genes with increasing altitude. *GJA4* and *FAP* are associated with vasomotion and fibrogenesis, and *GJA4* plays a key role in the vasomotion response, which can conducted from arterioles to larger arteries to promote topical blood circulation (Kim et al., 2018). *FAP* is highly upregulated in fibroblasts and can promote the deposition of fibers in the vessel wall (Niedermeyer et al., 2001; Jacob et al., 2012). The expression pattern of *GJA4* was clustered into profile 7 and *FAP* clustered into profile 4 (Figure 8A); it was, therefore, inferred that the increase in elastic fibers in the pulmonary artery of Tibetan sheep with altitude (Figure 3) is due to the increased expression of *GJA4* and *FAP* with increasing altitude.

Hb is a tetramer comprising two *HBB-* and two *HBA*-encoded polypeptide chains that transport oxygen and carbon dioxide and maintain blood acid–base balance, and the lung is an organ with considerable potential for hematopoiesis (Lefrancais et al., 2017). In this study, we found that *HBB* and *HBA1* in the lung of Tibetan sheep were one of the most prominent downregulated genes at H altitude compared to those at L altitude, and both were clustered into profile 0 (Figure 8A); this is contrary to that observed in previous studies. For example, the Hb concentration is higher in dogs at an altitude of 3000 m than in those at an altitude of 500 m (Gou et al., 2014); in the high-altitude sheep breeds, the Hb concentration and hematocrit (Hct) were higher than those of the low-altitude sheep breeds (Wei et al., 2016). To explain this paradox, we tested the HBB protein abundance in Tibetan sheep lungs using ELISA and found that the protein abundance of HBB first increased gradually and then decreased significantly with increasing altitude (*p* < 0.05) (Figure 9B), indicating that *HBB* is regulated during the translation process. The protein abundance of HBB is closer to the phenotype, and, in fact, the ELISA result is consistent with the results of Gou et al. (2014) and Wei et al. (2016); the highest protein abundance of HBB in this study was at the M altitude of 3500 m, which is equivalent to the high altitude in the studies of Gou et al. (2014) and Wei et al. (2016). The increase in Hb concentration with altitude enhances the oxygen-carrying capacity of the organism to overcome hypoxia; however, as altitude continues to increase, the increasing Hb concentration increases the viscous resistance of the blood, leading to pulmonary hypertension. Tibetan sheep may change their adaptation strategy when the Hb concentration reaches a harmful threshold, that is, decreasing Hb concentration but increasing relative lung weight, surface area of gas exchange, and capacity of the blood.

Among the DEGs between M and H altitudes, *APOLD1* and *CHL1* were one of the most prominent downregulated genes at H altitude and both were clustered into profile 5 (Figure 8A). *APOLD1* is associated with the formation of blood clots; *APOLD1* knockout mice promote the formation of blood clots after carotid artery injury compared to wild-type littermates (Aestro et al., 2020). The increased blood viscosity due to a high Hb concentration at M altitude makes blood clot formation easier; the upregulated expression of the *APOLD1* gene may play an inhibitory role in blood clot formation. It is notable that *APOLD1* was also one of the most significantly downregulated genes at L altitude compared to those at M altitude, and Hb concentration was lower at both L and H altitudes than at M altitude. *CHL1* is associated with homeostatic adaptation during hypoxia, compared with wild-type littermates; *CHL1* knockout mice exhibit an augmented ventilatory response after acute hypoxia (Huang et al., 2013); augmented ventilatory responses were also observed in Tibetan sheep (Wang et al., 2019) and Tibetans (Bigham and Lee, 2014) at high altitude. The significantly lower Hb concentration at H altitude weakens the oxygen-carrying capacity of the organism, but the enhanced ventilatory response can compensate to some extent; it is possible that the low expression of *CHL1* at H altitude is responsible for the enhanced ventilatory response.

Among the other DEGs between M and H altitudes, *COL1A1*, *COL1A2*, *COL3A1*, and *COL14A1* were one of the most prominent upregulated genes at H altitude, and they were clustered into profile 4 (Figure 8A). Collagen is the most common protein in animal organisms and is the basic component of blood vessels, which can bind with elastin to produce elastic recoil in lungs and arterial vessels (Van Doren, 2015). *COL1A1*, *COL1A2*, *COL3A1*, and *COL14A1* genes encode collagen and upregulate the expression at H altitude, resulting in increased vascular abundance and vasoconstrictive and vasodilatory capacity, which may also be a compensation for the reduced Hb concentration.

Functional enrichment analysis of DEGs between L and H altitudes revealed that fibrinolysis and blood vessel development were among the most enriched GO terms; these two terms were enriched by six DEGs (*HPSE*, *E2F8*, *SPARC*, *GATA1*, *GJA4*, and *FAP*). These genes have been reported to be essential for cardiovascular system and fiber development. For example, in addition to *HPSE*, *E2F8*, *GJA4*, and *FAP* mentioned above, *SPARC* upregulated at H altitude is involved in the basic cellular functions such as cell proliferation and differentiation and promotes microvascular remodeling (Wong and Sukkar, 2017). Other than that, the GO terms closely related to O_2_ transport and Hb metabolism were found herein, including gas transport, blood circulation, and hemoglobin metabolic process; these terms were also enriched by the DEGs between Tibetan chickens and Chahua chicken embryos under hypoxic and normoxic conditions (Zhang et al., 2020). In this study, these terms were enriched by eight DEGs (*ALAS2*, *GJA4*, *ACTG2*, *CNR1*, *VSNL1*, *Pol*, *HBB*, and *HBA1*); these genes play a key role in Hb metabolism and O_2_ transport, such as *ALAS2*, which is the first enzyme in the heme biosynthesis pathway in erythrocytes (Fujiwara and Harigae, 2013), which is downregulated at H altitude, may inhibit Hb synthesis, and may lower blood viscosity.

Regarding the DEGs between M and H altitudes, fibrillar collagen trimer, banded collagen fibril, collagen metabolic process, and vasculature development were among the most enriched GO terms. These terms were enriched by 10 genes (*COL1A1*, *COL1A2*, *COL2A1*, *COL3A1*, *COL5A1*, *COL5A3*, *LAMA1*, *LOXL2*, *MMP9*, and *SPARC*). Collagen fibers can positively regulate myogenesis (Nakajima et al., 2002) and lead to thickening of blood vessels. As expected, these genes, especially those of the collagen family (*COL1A1*, *COL1A2*, *COL2A1*, *COL3A1*, *COL5A1*, and *COL5A3*), were upregulated in H altitude. This may be one of the reasons for the vascular thickening at H altitude, allowing Tibetan sheep to compensate for the reduced Hb concentration. In addition, the GO terms regulation of blood vessel size, regulation of vasoconstriction, and blood circulation were enriched by four DEGs (*STC1*, *ADORA3*, *ALOX5*, and *FGG*) between L and M altitudes; these genes are associated with angiogenesis and remodeling. For example, *STC1* promotes (Law and Wong, 2013) and *ADORA3* inhibits (Rudich et al., 2015) angiogenesis and remodeling; the former is upregulated while the latter is downregulated at M altitude. These results may explain why pulmonary arterioles are thicker and have a larger cross-sectional area at M altitude than at L altitude.

The hematopoietic cell lineage pathway was the most significant pathway enriched by DEGs between L and M altitudes. This pathway was also significantly enriched when comparing the lung transcriptome of high-altitude yaks and low-altitude cattle (Xin et al., 2019; Ge et al., 2021). As the pO_2_ decreases with increasing altitude, indigenous animals that inhabit high altitude will increase the number of red blood cells within a reasonable range in order to maintain O_2_ homeostasis, which may be the reason for the hematopoietic cell lineage pathway being active. ECM–receptor interaction was one of the most significant pathways enriched by three upregulated genes (*LAMA1*, *COL1A1*, and *COL1A2*) at H altitude compared to those at M altitude; this pathway was also significantly enriched in the lungs of yaks at high altitude (Qi et al., 2019). Collagen encoded by *COL1A1* and *COL1A2* and laminin encoded by the involvement of *LAMA1* were the components of the ECM; the collagen is combined with elastin to form the main component of the pulmonary vasculature, while laminin is involved in a wide range of BPs such as cell adhesion, differentiation, and migration, allowing pulmonary vascular remodeling to occur with increasing altitude.

## Conclusion

In conclusion, this study found that the lungs of Tibetan sheep adapted differently to different altitudes. In addition to the continuous increase in the pulmonary artery volume, thickness, and elastic fiber content with altitude, Tibetan sheep at M altitude show an increase in the Hb concentration within a reasonable range, allowing for more efficient blood oxygen exchange. While at H altitude, they show a decrease in the Hb concentration and an increase in the surface area of gas exchange and capacity of the blood, allowing them to avoid pulmonary hypertension caused by increased Hb concentration. Other than that, some important DEGs related to angiogenesis (*FNDC1*, *HPSE*, and *E2F8*), vasomotion and fibrogenesis (*GJA4*, *FAP*, *COL1A1*, *COL1A2*, *COL3A1*, and *COL14A1*), and gas transport (*HBB*, *HBA1*, *APOLD1*, and *CHL1*) identified by transcriptomic data further validate these findings.

## Data Availability

The original contributions presented in this study are publicly available. These data can be found here: all the raw reads obtained in this study were uploaded to GenBank with accession numbers SRR16878507–SRR16878518.

## Footnotes

1
https://www.ncbi.nlm.nih.gov/assembly/GCF_002742125.1.

2
http://geneontology.org/.

3
http://www.genome.jp/kegg/.
